# Green Logistics and Health in OBRI Economies: Does Social Marketing Matter?

**DOI:** 10.3389/fpubh.2022.851344

**Published:** 2022-02-25

**Authors:** Lingyun Zhang, Hsu Ling Chang, Yin Dai, Alican Umut

**Affiliations:** ^1^Department of Marketing, School of Business, Wuchang University of Technology, Wuhan, China; ^2^Department of Accounting, Ling Tung University, Taichung City, Taiwan; ^3^The People's Bank of China, Guangzhou Branch, Guangzhou, China; ^4^Faculty of Economics and Administrative Sciences, Department of Economics and Finance, Nisantasi University, Istanbul, Turkey

**Keywords:** green logistics, social marketing, health, OBRI, GMM

## Abstract

In this study, our primary focus is to capture the impact of green logistics and social marketing on health outcomes in One Belt Road Initiative (OBRI) countries over the time period 2007–2019. Two estimation techniques, i.e., 2SLS and GMM, are employed to get the estimates of our variables. Findings of the 2SLS model confirmed the negative impact of green logistics on infant mortality in OBRI, European, MENA, and Asian countries. On the other side, the relationship between green logistics and life expectancy is positive in all the regions in 2SLS models. The other estimation technique also supports these findings, GMM, which confirmed the negative impact of green logistics on infant mortality and the positive impact of green logistics on life expectancy OBRI, European, MENA, and Asian economies. From these findings, we can conclude that green logistics helps to improve the health status of OBRI economies. Similarly, social marketing also improves the health status in OBRI and other regions in both models. Therefore, the governments and policymakers in respective economies should focus on the development of green infrastructure and logistics that, on one side, promote economic growth. However, on the other side, it helps improve environmental quality, which ultimately improves the OBRI economies' health status.

## Introduction

Green spaces and human health are positively linked. The benefits of green spaces on human health are many such as overall better health condition, the birth of a healthy child, better brain growth in infants and toddlers, better development of intellectual level in adults, enhanced mental health, reduced probability of serious and chronic sickness, and lower risk of early mortality (1–3). The path through which positive impacts of green spaces on human health are yet to be fully recovered, but minimizing stress or re-establishing cognition, improving physical movement, enlarging the biodiversity, and ecological microbial input is recommended by-products (4). According to the US Environmental Protection Agency (EPA), green spaces are those spaces or pieces of land that are “partly or completely closed with grass, trees, shrubs, or other vegetation,” which comprises “parks, community gardens, and cemeteries” (5). Though green spaces are reducing overtime in urban areas, their availability in these areas requires special attention and planning by higher authorities. Therefore, the quantity of green spaces available in different cities varies significantly, like 1.90 m2 to 52.0 m2 per person in Buenos Aires, Argentina, Curitiba, and Brazil, respectively.

Green infrastructure is the central focus of this study, the scope of which is broader than the green spaces (6–8). However, the definition of green infrastructure is not uniform, and it varies by a great deal. However, one definition is very common in the research circles, which stated that green infrastructure means many green spaces that are unified in one network that maintains the ecosystem's natural balance by preserving the environment (9–11). More precisely, green infrastructure may also include national parks, forests, orchards, gardens, and many other forms of green spaces that can benefit humans and positively impact the environment (1). In urban areas, green infrastructure is limited to the above-mentioned factors and includes a few others such as green roofs, road trees, and street plants that provide crucial support for the balance of the ecosystem in the cities areas without eating up too much land in the cities.

Another definition of green infrastructure was provided by the European Environment Agency, which states that green infrastructure includes a large number of environmental factors that work in different areas but are interconnected in an integrated network to preserve the environment (12). However, these factors are multifunctional; and they must surpass the green space in their utility and characteristics. For instance, a single tree in the mid of the urban area or a single patch of grass in the city may not be described as a green infrastructure without its contribution to resolving the major environmental challenges locally. Nonetheless, the operating functions of these spatial factors may vary considerably from a small single factor to a complete set of functional ecosystems that could prove beneficial in the development of green infrastructure in urban areas (13). This combination of green land and blue water areas can positively impact environmental quality and ultimately improve human health (3).

After clearing the readers' minds regarding green infrastructure, we are now in a position to describe the relationship between human health and environmental quality. Environmental models of health have progressed during recent times. These models have particularly focused on the positive impact of a clean environment on human health and quality of life (14). During the development of these models, the biosphere, landscape, and natural environment are considered as most important factors in improving citizens' health and quality of life (15, 16). Despite the importance of a clean environment in improving human health, this area is underexplored in the research spheres (17). Although the literature on the environmental quality and health status is limited, few good studies are available that have observed that apart from physical activity, social capital, and stress, natural landscapes and green spaces are also important contributors to improved human health. However, if we go through the present studies available in this regard, we know that these studies have focused more on the less important factors of ecosystems and ignored factors such as green logistics. Despite the numerous health benefits attached to green logistics, recent literature has overlooked its importance in improving human health (18). Ignoring the role of green logistics in improving people's health may put people's life at much greater risk.

Social marketing could be another important factor in improving people's health. However, health professionals often consider it an additional and less important factor in determining human health status. Nevertheless, in the last 20 years, social marketing has emerged as an important tool in serving society. With the evolution of mass communications and other techniques of knowledge diffusion, access to distant and remote communities has become easy. Messages that can affect social behavior and public choices and alter the mindset can spread more quickly and to a larger population. In the context of human health, the public service messages from government and civil society can reach as many people as possible within a limited time. Conversely, these channels can also use to spread messages that can prove detrimental to human health. The varying atmosphere of communication delivers a vital background for struggles to amend approach and conduct, of which social marketing is an example. Earlier literature is still ignoring the nexus between green logistics and health, but the direction is not clear among social marketing and health.

We consider that this is the first empirical study of its kind that tests the relationship of green logistics and social marketing with the health of OBRI economies. Hence, in this study, we have also included social marketing as an additional element to determine health quality apart from green logistics. This study provides us novelty nexus among green logistics and health for OBRI economies. This research work is not specific to a few countries' analysis, but relatively it contains OBRI group scenario. This study is a new effort to fill the research gap. This study distinguished the significance of green logistics and social marketing in OBRI economies and how it influences health. The increasing empirical evidence in favor of the links between green logistics, social marketing, and human health has attracted policymakers and development practitioners, who are strongly interested in the elaboration of healthy policies to achieve the SDGs. The research works on the effects of green logistics and social marketing on public health in OBRI countries are still significant in policymaking.

## Model and Methods

Last few decades, scholars have assessed the health benefits of green infrastructure (19, 20). As well, green logistics could favorably affect ecosystems as well as human health and well-being. Green logistics could contribute to population health via clean energy consumption and ecological networks. Similarly, social marketing also plays a positive role in gaining health benefits (21). Our study follows the literature (19, 22). To detect the impacts of green logistics and social marketing on health; we have used the following panel model forms:


$$\begin{array}{l} \text{Healt}{\text{h}}_{\text{it}}\;=\;\varphi_{0}+\;\varphi_{1}\text{G}{\text{L}}_{\text{it}}+\varphi_{2}\text{Interne}{\text{t}}_{\text{it}}+\varphi_{3}\text{H}{\text{E}}_{\text{it}}+\varphi_{4}\text{GD}{\text{P}}_{\text{it}}\\ \;+\;\varphi_{5}\text{FD}{\text{I}}_{\text{it}}+\alpha_{\text{i}}+\varepsilon_{\text{it}} \end{array}$$

Where are the health outcomes that depend on green logistics (*GL*), social marketing (via captured through the internet), health expenditure (HE), GDP per capita (GDP), and foreign direct investment (FDI). Green logistics and social marketing contribute to public health, thus estimates of φ_1_ &*φ*_2_ is expected to be positive. As for the effects of the control variables, health expenditure, GDP, FDI could be positive on health. While εi,t is the error term, but i and t represent country and time period, respectively. While *Health*_*it*−1_ is a lagged level of health outcomes in Equation (2). The extended panel model is:


$$\begin{array}{l} \text{Healt}{\text{h}}_{\text{it}}\;=\;\varphi_{0}+{\text{λ}}_{1}\text{Healt}{\text{h}}_{\text{it}-1}+\;\varphi_{1}\text{G}{\text{L}}_{\text{it}}+\varphi_{2}\text{Interne}{\text{t}}_{\text{it}}\\ \;+\;\varphi_{3}\text{H}{\text{E}}_{\text{it}}+\varphi_{4}\text{GD}{\text{P}}_{\text{it}}+\varphi_{5}\text{FD}{\text{I}}_{\text{it}}+\alpha_{\text{i}}+\varepsilon_{\text{it}} \end{array}$$

In our panel models, green logistics is the endogenous regressor and principal variable. Thus, firstly, we estimate the econometric model with an endogenous variable by using the 2SLS, which fixed the problem of endogeneity in the panel model. The second method to examine the relationship between green logistics and social marketing on health is a panel method (GMM). Previous standard literature has the same econometric approaches are employed for health outcomes (23, 24). We employ a generalized method of moments (GMM) for panel data developed by Arellano & Bond (25), which is used to resolve the issue of serial correlation, heteroskedasticity, and endogeneity. The GMM is a more valuable approach than 2SLS in panel data, but both are instrumental variables estimation approaches. These issues commonly exist in panel data analysis. This method is a particularly useful choice when the cross-section (i) is larger than the data span (*t*). Since our panel data consists of 45 countries and a relatively small time period of 13 years (from 2007 to 2019), the GMM estimator is a suitable technique. Normally two types of the GMM estimator exist, namely two-step and one-step GMM. The two-step estimator attains better results regarding the econometric problems than the one-step estimator (26). Thus, we can use a two-step GMM estimator in our model.

## Data

The study aims to investigate the impact of green logistics and social marketing on health outcomes in the case of OBRI economies for the period 2007–2019. Table 1 displays the detailed information regarding symbols and definitions of all variables. Health outcomes are measured through two proxies: infant mortality rate and life expectancy. The infant mortality rate is measured in per 1,000 live births, and life expectancy is taken as life expectancy at birth, in total years. However, the green logistics index and social marketing are focused variables of the study. Social marketing is measured by individuals using the internet in percent of the total population. Health expenditures (in the percentage of GDP), GDP per capita (measured as constant 2015 US$), and FDI (measured as foreign direct investment inflows in percent of GDP) are incorporated as control variables. Only the GDP per capita variable has been transformed logarithmically. Data on all these variables have been sourced from the World Bank, and descriptive analysis is reported in Table 2.

**Table 1:** Variable's definitions.

**Variables**	**Symbol**	**Definitions**
Infant mortality	IM	Mortality rate, infant (per 1,000 live births)
Life expectancy	LE	Life expectancy at birth, total (years)
Green logistics	GL	Green logistics index
Internet users intensity	Internet	Individuals using the internet (% of the population)
Health expenditure	HE	Current health expenditure (% of GDP)
GDP per capita	GDP	GDP per capita (constant 2015 US$)
Foreign direct investment	FDI	Foreign direct investment inflows(% GDP)

**Table 2:** Descriptive statistics.

		**IM**	**LE**	**GL**	**Internet**	**HE**	**GDP**	**FDI**
**OBRI**	Obs	585	585	585	585	585	585	585
	Mean	15.9	73.1	2.87	46.7	5.57	8.63	4.29
	Std. Dev.	13.2	3.88	0.46	25.9	1.86	0.98	7.21
	Min	1.70	64.4	1.80	0.93	1.94	6.55	−41.5
	Max	74.1	83.5	4.19	96.1	11.4	11.0	54.6
**Europe**	Obs	210	210	210	210	210	210	210
	Mean	7.83	74.5	2.94	60.4	6.82	9.04	4.57
	Std. Dev.	6.50	2.83	0.36	19.7	1.57	0.67	7.47
	Min	1.70	68.2	2.14	6.02	1.94	7.63	−41.5
	Max	39.3	81.3	3.68	94.4	11.4	10.0	54.6
**MENA**	Obs	117	117	117	117	117	117	117
	Mean	19.4	73.5	2.71	42.9	5.81	8.63	2.42
	Std. Dev.	11.3	4.37	0.41	24.0	1.60	0.89	2.94
	Min	3.00	64.4	2.03	0.93	2.69	6.98	−4.34
	Max	49.3	82.8	4.19	95.7	9.50	10.5	15.1
**Asia**	Obs	247	247	247	247	247	247	247
	Mean	21.5	71.6	2.89	36.2	4.33	8.27	4.93
	Std. Dev.	14.8	3.93	0.53	26.2	1.36	1.10	8.20
	Min	2.10	64.4	1.80	1.80	2.28	6.55	−37.1
	Max	74.1	83.5	4.19	96.1	8.51	11.0	43.9

## Results and Discussion

As the study explores the impact of green logistics and social marketing on health in OBRI economies, Table 2 reports the findings of descriptive statistics. The study is also considering the OBRI connecting regions such as Europe, MENA, and Asia, for investigating the effect of green logistics on health outcomes. The study gets assistance from two econometric approaches, i.e., 2SLS and GMM, for empirical investigation. Table 3 displays the health outcomes of the 2SLS model, and Table 4 provides the coefficient estimates of health outcomes under the GMM approach.

**Table 3:** Health outcomes (2SLS).

	**Infant mortality**				**Life expectancy**			
	**OBRI**	**Europe**	**MENA**	**Asia**	**OBRI**	**Europe**	**MENA**	**Asia**
GL	−5.181^**^	−1.336^***^	−0.516^***^	−1.042	0.619^**^	0.120^***^	0.055^***^	2.730
	2.481	−0.329	−0.159	−2.625	−0.254	−0.027	0.016	−2.906
Internet	−0.019^*^	−0.029^***^	−0.082^***^	−0.026	0.009^***^	0.005^**^	0.006^***^	0.016^***^
	−0.011	−0.002	−0.001	−0.127	−0.001	0.002	0.001	−0.005
HE	−0.145	−0.011	−0.007	−0.494	0.013	0.004^**^	−0.002	0.101
	−0.125	−0.029	−0.020	−1.995	−0.013	−0.002	−0.002	−0.391
GDP	−1.964^*^	−0.603^***^	−0.472^***^	−2.520^**^	0.188^*^	0.003	0.043^***^	0.573^*^
	−1.087	−0.227	−0.142	−1.056	−0.101	−0.018	−0.014	−0.359
FDI	0.017	−0.001	−0.003	−0.058	−0.002	0.000	0.00134^*^	0.012
	−0.014	−0.002	−0.008	−0.240	−0.001	0.000	−0.001	−0.047
Constant	4.097	11.44^***^	5.839^***^	24.03	4.294^***^	3.930^***^	4.050^***^	0.825
	−4.116	−2.022	−0.955	−63.14	−0.433	−0.164	−0.0965	−12.36
Observations	585	221	117	247	585	221	117	247
Country	45	17	9	19	45	17	9	19

*** *p < 0.01*.

** *p < 0.05*.

* *p < 0.1*.

**Table 4:** Health outcomes (GMM).

	**Infant mortality**				**Life expectancy**			
	**OBRI**	**Europe**	**MENA**	**Asia**	**OBRI**	**Europe**	**MENA**	**Asia**
L. infant mortality	0.958^***^	0.975^***^	0.968^***^	0.949^***^				
	−0.005	−0.011	−0.012	−0.003				
L. life expectancy					0.854^***^	0.766^***^	0.896^***^	0.917^***^
					−0.017	−0.034	−0.013	−0.014
GL	−0.007^**^	0.004	−0.009^***^	−0.181^***^	0.060^*^	0.015^**^	0.001	0.051^*^
	−0.003	−0.006	−0.003	−0.057	0.033	−0.006	0.002	0.030
Internet	−0.012	−0.023^**^	−0.011	−0.004^***^	0.083^***^	0.029^***^	0.635^*^	0.002^*^
	0.014	0.011	0.012	−0.001	−0.023	0.011	0.002	−0.001
HE	−0.002^**^	−0.004^**^	0.001	−0.023	0.023^***^	0.010	0.008	0.039^*^
	−0.001	−0.002	−0.001	−0.018	−0.007	0.020	0.012	−0.022
GDP	−0.028^***^	−0.025	−0.028^***^	−0.222^**^	0.248^***^	0.001	0.004^***^	0.058
	−0.005	−0.017	−0.007	−0.092	−0.091	−0.004	−0.001	−0.090
FDI	0.011	0.030	0.034	−0.003^***^	0.011	0.023	0.031	0.123
	0.021	0.025	0.344	−0.001	−0.021	0.033	0.123	−0.231
Constant	0.333^***^	0.231	0.320^***^	2.491^***^	8.689^***^	0.991^***^	0.412^***^	5.684^***^
	−0.053	−0.178	−0.073	−0.77	−1.054	−0.135	−0.056	−0.889
Observations	495	187	99	209	495	187	99	209
Country	45	17	9	19	45	17	9	19
Sargan test	0.523	0.452	0.630	0.403	0.324	0.238	0.256	0.356

*** *p < 0.01*.

** *p < 0.05*.

* *p < 0.1*.

Health outcomes of the 2SLS model reveal that green logistics exert a significant and negative impact on infant mortality in OBRI, Europe, and MENA economies, confirming that green logistics tend to reduce infant mortality rates significantly. Coefficient estimates reveal that in response to a 1% upsurge in green logistics, the infant mortality rate is reduced by 5.181% in OBRI, 1.336% in Europe, and 0.516% in MENA countries. However, green logistics produce an insignificant impact on the infant mortality rate in case of Asia. In terms of control variables, the internet produces a significant and negative impact on infant mortality rate, revealing that due to a 1% increase in the internet, infant mortality rate declines in OBRI, Europe, and MENA economies by 0.019, 0.029, and 0.082%, respectively. However, health expenditures and FDI have no impact on infant mortality in OBRI and connected regions as the respective coefficient estimates are statistically insignificant. GDP exerts a significant and negative impact on infant mortality, confirming that a 1% upsurge in GDP reduces the infant mortality rate by 1.964% in OBRI, 0.603% in Europe, 0.472% in MENA, and 2.520% in Asia.

In the case of the life expectancy model, findings of the 2SLS approach demonstrate that green logistics have a significant and positive impact on life expectancy in the case of OBRI, MENA, and Europe, confirming that life expectancy tends to increase due to an increase in green logistics. Findings show that a percent increase in green logistics results in increasing life expectancy by 0.619% in OBRI, 0.120% in Europe, and 0.055% in the case of MENA economies. This finding is also consistent with Nieuwenhuijsen (3), who noted that green logistics effectively reduces carbon emissions and improves health outcomes. Green logistics improve environmental efficiency and is mainly related to urban sectors. Green logistics is the interrelated network of green technology that protects natural environmental functions and values and delivers supplementary benefits to societies. Green logistics as an environmental adaptation strategy has the prospective to mitigate many dangers to human health outcomes posed by environmental change, in association with mental and physical well-being and health (27).

Additionally, green logistics provide operational cooling against climatic temperatures, specifically in urban zones. Various studies claimed that green logistics can indirectly mitigate stress levels and can reduce the adverse health effects of environmental pollution (28). Access to green logistics is attached with better-quality recovery from illness. Green logistics may help control the spread of certain diseases such as allergy rates, respiratory diseases, obesity, and body mass indexes, which improves life expectancy and reduces mortality rates. Our green logistics finding is also supported by Agyabeng-Mensah, et al. (29), who infers that green logistics contributes significantly to the protection of the environment. Moreover, green logistics conserves the reliability of the environmental system and can deliver a physical basis for environmental networks. The connection between public health and environmental health is the set of environmental services delivered by Green logistics.

Internet use also exerts a significant and positive impact on life expectancy. A 1 percent increase in internet use results in increasing life expectancy by 0.009, 0.005% in Europe, 0.006% in MENA, and 0.016% in Asia. This finding is supported by Terblanche-Smit and Terblanche (22), who argued that social marketing could target the environment, communities, and individuals to support and improve human health outcomes within the public health framework. The social marketing approach reveals that publicity contributes significantly to attaining the objectives of the program and plays a significant role in enhancing the life quality of the targeted zones. It is highlighted that the basic aim of the social marketing approach is to change the behavior of society for the sake of health improvements.

Health expenditure produces a significant and positive impact on life expectancy only in Europe, revealing that a 1 percent rise in health expenditures improves life expectancy by 0.004% in the region. GDP also exerts a significant and positive impact on life expectancy in all selected regions except Europe. Coefficient estimates reveal that a 1% upsurge in GDP increases life expectancy by 0.188% in OBRI, 0.043% in MENA, and 0.573% in Asia, respectively. FDI results in significantly increased life expectancy only in the case of the MENA region with a magnitude of 0.013%.

The health outcomes of the GMM approach reveal that green logistics tends to reduce the infant mortality rate in all selected regions except Europe and significantly improves life expectancy in all the selected regions except MENA. Coefficient estimates confirm that a 1% increase in green logistics result in reducing the infant mortality rate by 0.007% in OBRI, 0.009% in MENA, and 0.181% in Asia, however, and increases life expectancy by 0.060% in OBRI, 0.015% in Europe, and 0.051% in Asia. Internet use reduces infant mortality rates in Europe and Asia and significantly improves life expectancy in all selected regions. Health expenditures tend to reduce the infant mortality rate in OBRI and Europe and improve life expectancy in the case of OBRI and Asia regions. GDP declines infant mortality rate in all selected regions except Europe. However, it improves life expectancy in OBRI and MENA regions. However, FDI only reduces the infant mortality rate in Asia and significantly impacts life expectancy.

## Conclusion and Implications

The health status of the nation is highly dependent on the economic development of a country. In highly developed economies, more resources can be diverted toward health facilities. As a result, these economies enjoy a low mortality rate and high life expectancy compared to the developing economies. However, in recent times the concept of sustainable development has taken over the traditional concept of economic development. To achieve economic development, myriad factors have been analyzed by empirics and researchers. Green infrastructure and green logistics got popular most recently; however, their relationship with sustainable economic development is still an area that needs further exploration. The health status of the nation also improved along with economic development. It means the factors that affect economic development may also affect the health status of the nation. Therefore, in this study, our primary focus is to capture the impact of green logistics and social marketing on health in OBRI countries. Two estimation techniques, i.e., 2SLS and GMM, are employed to get the estimates of our variables.

Findings of the 2SLS model confirmed the negative impact of green logistics on infant mortality of OBRI, Europe, MENA, and Asian countries. On the other side, the relationship between green logistics and life expectancy is positive in all the regions in 2SLS models. These findings are also supported by the other estimation technique GMM, which confirmed the negative impact of green logistics on infant mortality and the positive impact of green logistics on life expectancy OBRI, European, MENA, and Asian economies. From these findings, we can conclude that green logistics helps to improve the health status of OBRI economies. Similarly, the internet estimates also improve the health status in OBRI and other regions in both models.

The results alone don't provide any benefit unless we provide policy guidelines based on these findings. Therefore, we provided important policy implications for all stakeholders. The green logistic has improved the health status of the OBRI economies. Therefore, the governments and policymakers in respective economies should focus on the development of green infrastructure and logistics that, on one side, promote economic growth. However, on the other side, it helps improve environmental quality, which ultimately improves the OBRI economies' health status. Moreover, the policymakers should try to get the correct estimates of the direct benefits of green logistics on health status. Further, the spread and use of the internet should be increased in the health sector that would ultimately disseminate health information and services at every corner of the country.

This study cannot examine the impact of green logistics and social marketing on human wellbeing. Future studies can scrutinize the transmission channels and the relationship between green logistics, social marketing, and human wellbeing. The comparative analysis will be more helpful in explaining the health and human wellbeing difference in developed and developing economies. Furthermore, other indicators of health and human wellbeings, such as environmental issues, energy consumption, and education can be included to extend the empirical analysis.

## Data Availability Statement

Publicly available datasets were analyzed in this study. This data can be found here: https://data.worldbank.org/.

## Author Contributions

LZ and HC: conceptualization, software, data curation, and writing-original draft preparation. YD: methodology, writing-reviewing, and editing. AU: visualization and investigation. All authors contributed to the article and approved the submitted version.

## Conflict of Interest

The authors declare that the research was conducted in the absence of any commercial or financial relationships that could be construed as a potential conflict of interest.

## Publisher's Note

All claims expressed in this article are solely those of the authors and do not necessarily represent those of their affiliated organizations, or those of the publisher, the editors and the reviewers. Any product that may be evaluated in this article, or claim that may be made by its manufacturer, is not guaranteed or endorsed by the publisher.
